# Gene Transfer of Skeletal Muscle-Type Myosin Light Chain Kinase via Adeno-Associated Virus 6 Improves Muscle Functions in an Amyotrophic Lateral Sclerosis Mouse Model

**DOI:** 10.3390/ijms23031747

**Published:** 2022-02-03

**Authors:** Ryohei Oya, Osamu Tsukamoto, Tatsuro Hitsumoto, Naoya Nakahara, Chisato Okamoto, Ken Matsuoka, Hisakazu Kato, Hidenori Inohara, Seiji Takashima

**Affiliations:** 1 Department of Medical Biochemistry, Osaka University Graduate School of Medicine/Frontier Biosciences, Osaka 565-0871, Japan; roya@ent.med.osaka-u.ac.jp (R.O.); hituhituhitu@gmail.com (T.H.); saiseikaichisato@gmail.com (C.O.); kmatsuoka@medbio.med.osaka-u.ac.jp (K.M.); katohisa@medbio.med.osaka-u.ac.jp (H.K.); takasima@cardiology.med.osaka-u.ac.jp (S.T.); 2 Department of Otorhinolaryngology Head and Neck Surgery, Osaka University Graduate School of Medicine, Osaka 565-0871, Japan; hinohara@ent.med.osaka-u.ac.jp; 3 Department of Molecular Physiology, The Jikei University School of Medicine, Tokyo 105-8461, Japan; nkhr@jikei.ac.jp

**Keywords:** adeno-associated virus 6, myosin light chain kinase, muscle function, amyotrophic lateral sclerosis, gene therapy

## Abstract

Amyotrophic lateral sclerosis (ALS) is a neurodegenerative disease that shows progressive muscle weakness. A few treatments exist including symptomatic therapies, which can prolong survival or reduce a symptom; however, no fundamental therapies have been found. As a therapeutic strategy, enhancing muscle force is important for patients’ quality of life. In this study, we focused on skeletal muscle-specific myosin regulatory light chain kinase (skMLCK), which potentially enhances muscle contraction, as overexpression of skMLCK was thought to improve muscle function. The adeno-associated virus serotype 6 encoding skMLCK (AAV6/skMLCK) and eGFP (control) was produced and injected intramuscularly into the lower limbs of SOD1^G37R^ mice, which are a familial ALS model. AAV6/skMLCK showed the successful expression of skMLCK in the muscle tissues. Although the control did not affect the muscle force in both of the WT and SOD1^G37R^ mice, AAV6/skMLCK enhanced the twitch force of SOD1^G37R^ mice and the tetanic force of WT and SOD1^G37R^ mice. These results indicate that overexpression of skMLCK can enhance the tetanic force of healthy muscle as well as rescue weakened muscle function. In conclusion, the gene transfer of skMLCK has the potential to be a new therapy for ALS as well as for other neuromuscular diseases.

## 1. Introduction

Amyotrophic lateral sclerosis (ALS) is a neurodegenerative disease that shows progressive muscle weakness due to the selective loss of both the upper and lower motor neurons [1,2,3]. ALS is divided into two groups: familial ALS (fALS) and sporadic ALS (sALS); fALS occupies 10% of all ALS cases [4]. Several genes have been reported as the ALS-causing or associating genes [5,6]. Among them, the mutations in the Cu/Zn superoxide dismutase 1 (SOD1) gene are the most studied [6,7], with the frequency of SOD1 mutations reported to be from 12% to 23% and from 0% to 7% in patients with fALS and sALS, respectively [6]. Transgenic mice expressing the mutant SOD1^G93A^ (glycine substituted to alanine at position 93) are well recognized as an ALS mouse model because they develop motor neuron disorders similar to that of a human ALS [4,8]. Conversely, the SOD1 knockout mouse has not shown any motor neuron disorders [9]. Therefore, the toxic gain of function of mutant SOD1 has been proposed as an important cause of fALS [10]. Indeed, the mutant SOD1 has been shown to exert its toxicity through misfolding and aggregation [11,12,13], and a recombinant monoclonal antibody that selectively binds to misfolded SOD1 has been shown to reduce misfolded SOD1, thus potentially delaying the onset and slowing the progression of ALS in mice [14].

Although a few treatments exist, including symptomatic therapies that can prolong survival or reduce a symptom [15], these cannot sufficiently rescue ALS patients from severe motor deficits and fatal paralysis. Despite such treatments, most patients die due to respiratory failure within 3–5 years of disease onset [1,2]. Recently, gene therapies for various neurodegenerative diseases, including fALS, have been under development, with some clinical trials underway [16]. However, gene therapies targeting the disease-causing gene mutations are applicable only for patients with the mutations and not for patients with unknown etiology. Therefore, new approaches for treating patients with ALS are required.

Rehabilitation is required for ALS patients principally aimed to preserve their remaining muscle functions for better quality of life (QOL). If the muscle contractility of the affected muscles in ALS patients could be improved beyond the rehabilitation as well as disease progression, it should also greatly improve patients’ QOL. Thus, the strategy to improve the muscle contraction will be one of the critical options for ALS treatment, and the molecules that can enhance muscle contraction are intended to be a new therapeutic target for ALS. The contractile force of striated muscle is produced by the interaction between thick myosin filaments and thin actin filaments [17]. Myosin II is a motor protein that is composed of two myosin heavy chains, two regulatory light chains (RLC), and two essential light chains. Importantly, RLC phosphorylation has been known to be a physiological modulator of the developing force in muscle cells [18]. RLC is phosphorylated mainly by myosin regulatory light chain kinase (MLCK) that is catalytically activated by the binding of Ca^2+^/calmodulin (CaM) complex. Among the three types of MLCK proteins, the skeletal muscle-type MLCK (skMLCK), encoded by the *MYLK2* gene, is expressed specifically in the skeletal muscle, and therefore, the activity or expression level of the skMLCK modulates the contractility in the skeletal muscle [18,19,20]. Indeed, a previous study has demonstrated that the gene ablation of *MYLK2* showed the attenuated isometric twitch tension after a brief tetanic stimulation (at 150 Hz for 2 s), a phenomenon called post-tetanic potentiation (PTP), in mice [21]. Another study also reported that overexpression of the skMLCK protein in the skeletal muscles upregulated the phosphorylation level of the RLC and enhanced the potentiation of successive isometric twitches during low-frequency stimulation, a phenomenon called staircase potentiation, in mice [22]. These studies indicated that the upregulation of the phosphorylation level of RLC could increase the developing force of the skeletal muscles. Accordingly, we hypothesized that the increased RLC phosphorylation by the gene therapy that overexpresses skMLCK could rescue the muscle weakness in ALS patients regardless of the variety of gene mutation, which can be a new therapeutic option of ALS treatments.

This study aims to investigate whether the skMLCK gene transfer into the skeletal muscles of the SOD1^G93A^ mice via AAV vector can improve muscle function.

## 2. Results

### 2.1. Generation of AAV6 Vectors Expressing skMLCK

**Figure 1:**
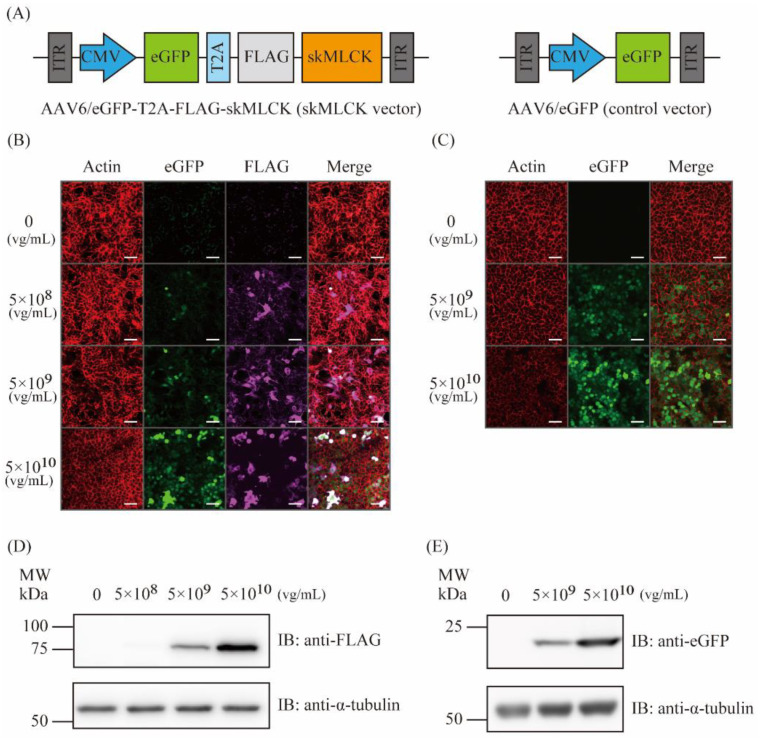
The observed expression of target protein in 293T cells by AAV vectors. (**A**) The scheme of the produced viral vectors. The left panel shows the structure of the skMLCK vector, which contains the eGFP, T2A peptide, FLAG tag, and skMLCK gene downstream of the cytomegalovirus (CMV) promoter. The right panel shows the structure of the control vector, which contains only the eGFP gene downstream of the CMV promoter. (**B**) Immunofluorescence staining against actin (phalloidin), eGFP, and FLAG in 293T cells infected by the skMLCK vector (0, 5 × 10^8^, 5 × 10^9^, and 5 × 10^10^ vg/mL); scale bar: 50 μm. (**C**) Immunofluorescence staining against actin (phalloidin) and eGFP in 293T cells infected by the control vector (0, 5 × 10^9^ and 5 × 10^10^ vg/mL); scale bar: 50 μm. (**D**) Immunoblot analysis of the skMLCK proteins using anti-FLAG antibodies in 293T cells infected by the skMLCK vector (0, 5 × 10^8^, 5 × 10^9^, and 5 × 10^10^ vg/mL). α-tubulin was used as a loading control. (**E**) Immunoblot analysis of the eGFP proteins using anti-eGFP antibodies in 293T cells infected by the control vector (0, 5 × 10^9^ and 5 × 10^10^ vg/mL). α-tubulin was used as a loading control.

In this study, the AAV6-eGFP-T2A-skMLCK (AAV6 skMLCK vector) and AAV6-eGFP (AAV6 control vector) vectors were first generated (Figure 1A). Two days after infection of the AAV6 vectors in HEK293 cells, the expression of the target proteins was examined using immunofluorescence staining and immunoblotting. Both immunofluorescence staining (Figure 1B,C) and immunoblotting (Figure 1D,E) demonstrated successful expressions of the target genes in a dose-dependent manner.

### 2.2. The Intramuscular Injection of the Control AAV6 Vector Did Not Affect the Muscle Force Produced by Twitch and Tetanic Stimulations in Wild-Type Mice

**Figure 2:**
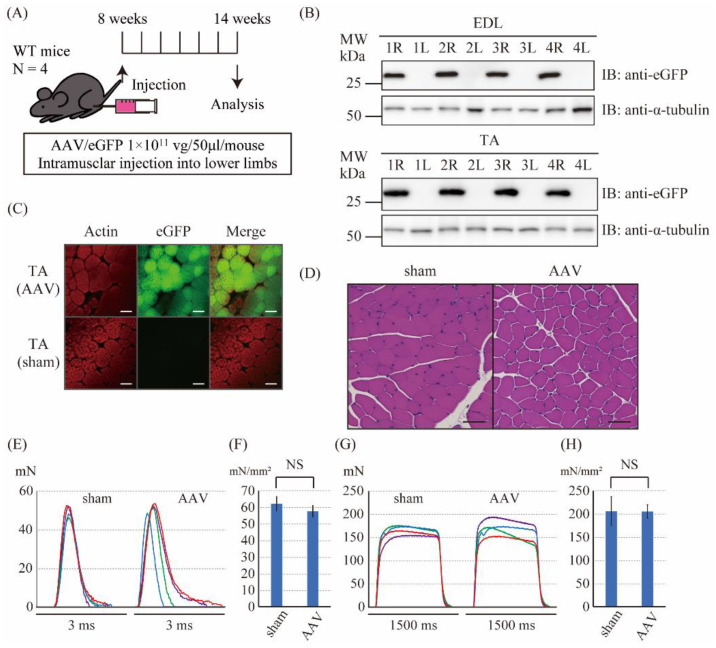
The gene transfer to wild-type mice using the control vector. (**A**) The scheme of the injection of the control vector. The control vector (1 × 10^11^ vg/mouse) was injected into the right lower limb, and saline was injected into the left lower limb at the age of 8 weeks. A series of analysis was performed after 6 weeks. (**B**) Immunoblot analysis of the eGFP proteins in the extensor digitorum longus (EDL) (upper panel) and tibialis anterior (TA) muscles (lower panel). R (right side) is the AAV-injected side, and L (left side) is the sham side. α-tubulin was used as a loading control. (**C**) Immunofluorescence staining against actin (phalloidin) and eGFP in the TA muscle; scale bar: 50 μm. (**D**) Hematoxylin and eosin (HE) stain of the TA muscle; scale bar: 50 μm. (**E**) The twitch force of the isolated EDL muscle of the sham and the AAV sides. The data shown in the same color were from the same mouse. (**F**) The normalized twitch force calculated from E. No significant difference was observed between the sham (62.1 ± 4.38 mN/mm^2^) and the AAV sides (57.6 ± 3.40 mN/mm^2^). (**G**) The tetanic force of isolated EDL muscle of the sham and the AAV sides. The data shown in the same color were from the same mouse. (**H**) The normalized tetanic force calculated from G. No significant difference was observed between the sham (205.6 ± 31.7 mN/mm^2^) and the AAV sides (204.9 ± 14.7 mN/mm^2^).

To examine whether the AAV-control vector can work in vivo, the control AAV vector was injected intramuscularly into the right lower limb (AAV side), and saline was injected into the left lower limb (sham side) of WT mice (Figure 2A). Six weeks after the injection, the expression of eGFP protein in the lower limbs was examined. Immunoblotting of the extensor digitorum longus (EDL) and tibialis anterior (TA) muscle tissues showed the expression of eGFP in the limb of AAV6 side but not in the limb of the sham side (Figure 2B). Immunofluorescence staining of TA muscle tissues also showed the expression of eGFP in the limb of AAV6 side but not in the limb of the sham side (Figure 2C). Hematoxylin and eosin (HE) stains of T.

A muscle tissues did not show the obvious differences in shapes of cells among the AAV and sham sides (Figure 2D). Thus, we successfully expressed the eGFP proteins in the muscles of the lower limbs by intramuscular injection of the control AAV6 vector in the WT mice. Next, we investigated the effects of the control AAV6 vector injection on the muscle force production in the EDL muscle of the WT mice. The cross-sectional area, which was calculated from the weight and length of isolated EDL muscles, showed no significant difference in each side (Supplementary Material). No significant differences in the normalized twitch force was observed between the sham and the AAV sides (62.1 ± 4.38 mN/mm^2^ vs. 57.6 ± 3.40 mN/mm^2^, respectively, N = 4, *p* = 0.15, Figure 2E,F). Furthermore, no significant differences in the normalized tetanic force were observed between the sham and the AAV sides (205.6 ± 31.7 mN/mm^2^ vs. 204.9 ± 14.7 mN/mm^2^, respectively, N = 4, *p* = 0.97, Figure 2G,H). These results indicated that the control AAV6 vector injection did not affect the muscle force generation in the WT mice.

### 2.3. The Intramuscular Injection of the skMLCK Vector Increased the Tetanic Force but Not the Twitch Force of Muscles in WT Mice

**Figure 3:**
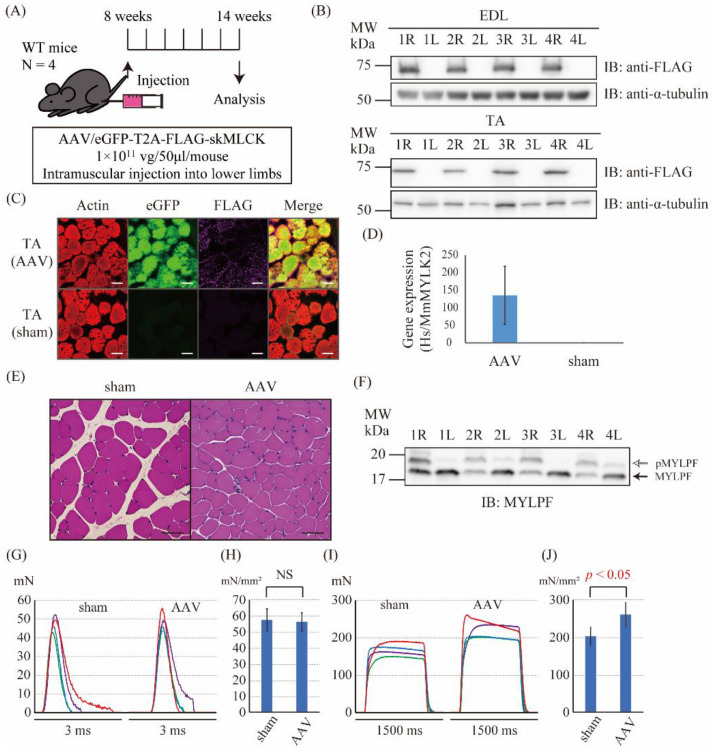
The gene transfer to wild-type mice using the skMLCK vector. (**A**) The scheme of the injection of the skMLCK vector. The control vector (1 × 10^11^ vg/mouse) was injected into the right lower limb, and saline was injected into the left lower limb at the age of 8 weeks. A series of analysis was performed after 6 weeks. (**B**) Immunoblot analysis of the FLAG peptide in the extensor digitorum longus (EDL) (upper panel) and tibialis anterior (TA) muscles (lower panel). R (right side) is the AAV-injected side, and L (left side) is the sham side. α-tubulin was used as a loading control. (**C**) Immunofluorescence staining against actin (phalloidin), eGFP, and FLAG in the TA muscle; scale bar: 50 μm. (**D**) Gene expression of human (Hs) *MYLK2* gene in the TA muscle. Gene expression is demonstrated as the ratio of *HsMYLK2* to mouse (Mm) *MYLK2*. (**E**) Hematoxylin and eosin (HE) stain of the TA muscle; scale bar: 50 μm. (**F**) The Phos-tag PAGE of EDL muscle. The upper band showed the phosphorylated MYLPF protein (pMYLPF, white arrow) and the lower band showed the non-phosphorylated MYLPF protein (black arrow). R (right side) is the AAV-injected side, and L (left side) is the sham side. (**G**) The twitch force of the isolated EDL muscle of the sham and the AAV sides. The data shown in the same color were from the same mouse. (**H**) The normalized twitch force calculated from G. No significant difference was observed between the sham (57.4 ± 7.09 mN/mm^2^) and the AAV sides (56.3 ± 5.65 mN/mm^2^). (**I**) The normalized tetanic force of the isolated EDL muscle of the sham and the AAV sides. The data shown in the same color were from the same mouse. (**J**) The normalized tetanic force calculated from I. The normalized tetanic force of the AAV sides (260.6 ± 32.5 mN/mm^2^) was significantly greater than that of the sham sides (202.1 ± 24.2 mN/mm^2^, *p* = 0.028).

Next, to examine whether the AAV6-skMLCK vector could work in vivo, the AAV6-skMLCK vector was injected intramuscularly into the right lower limb (AAV side) and the saline was injected into the left lower limb (sham side) of the WT mice (Figure 3A). Six weeks after the injection, we examined the expression of eGFP and FLAG-skMLCK proteins in the lower limbs. Immunoblotting of EDL and TA muscle tissues showed the expression of FLAG proteins in the limb of AAV6 side but not in the limb of sham side (Figure 3B). Immunofluorescence staining of the TA muscle tissues also showed the expression of eGFP and FLAG proteins in the limb of the AAV6 side but not in the limb of the sham side (Figure 3C). Droplet digital PCR (ddPCR) showed that the human (Hs) *MYLK2* gene expressed only in the AAV-injected sides. The ratio of *HsMYLK2* gene expression to mouse (Mm) *MYLK2* in the AAV side was 135.3 ± 82.9 (Figure 3D, N = 4). HE stains of the TA muscle tissues did not show the obvious differences in shapes of cells among the AAV and sham sides (Figure 3E). Thus, we successfully overexpressed the skMLCK protein in the muscles of the lower limbs by intramuscular injection of the control AAV6 vector in the WT mice. Moreover, Phos-tag PAGE was performed to investigate the ratio of phosphorylated RLC and non-phosphorylated RLC. The level of the phosphorylated skeletal RLC known as MYLPF was significantly upregulated in the AAV sides by Phos-tag PAGE (Figure 3F). Next, we investigated the effect of the AAV6-skMLCK vector injection on the muscle force production in the EDL muscle of the WT mice. The cross-sectional area of the isolated EDL muscle showed no significant difference in each side (Supplementary Material). There were no significant differences in the normalized twitch force between the sham and the AAV sides (57.4 ± 7.09 mN/mm^2^ vs. 56.3 ± 5.65 mN/mm^2^, respectively, N = 4, *p* = 0.82, Figure 3G,H). In contrast, the normalized tetanic force of the AAV-injected sides was significantly larger than that of the sham sides (260.6 ± 32.5 mN/mm^2^ vs. 202.1 ± 24.2 mN/mm^2^, respectively, N = 4, *p* = 0.028, Figure 3I,J). Thus, the overexpression of the skMLCK protein by the AAV6-skMLCK vector followed by phosphorylation of MYLPF increased the tetanic force but not the twitch force of muscles in WT mice.

### 2.4. The Intramuscular Injection of the Control Vector Did Not Affect the Muscle Force Produced by Twitch and Tetanic Stimulations in SOD1^G93A^ Mice

**Figure 4:**
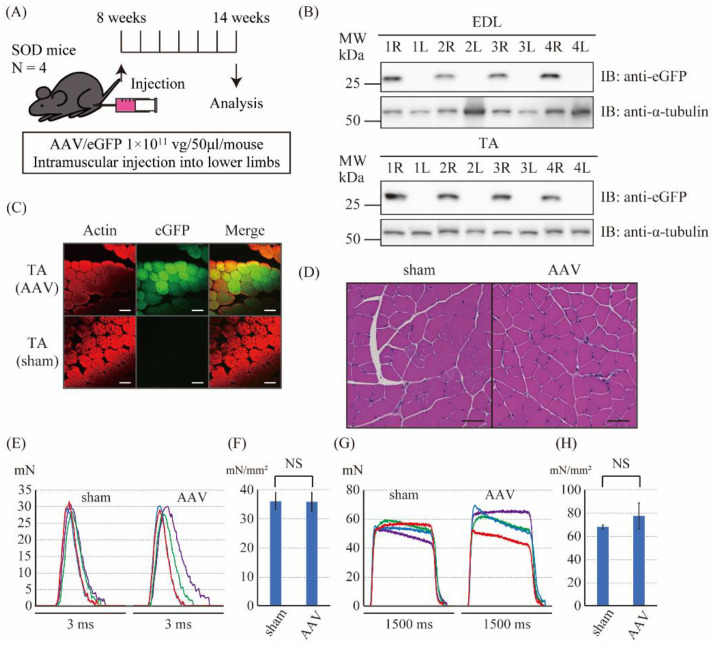
The gene transfer to SOD1 mice using the control vector. (**A**) The scheme of the injection of the control vector. The control vector (1 × 10^11^ vg/mouse) was injected into the right lower limb, and saline was injected into the left lower limb at the age of 8 weeks. A series of analysis was performed after 6 weeks. (**B**) Immunoblot analysis of the eGFP proteins in the extensor digitorum longus (EDL) (upper panel) and tibialis anterior (TA) muscles (lower panel). R (right side) is the AAV-injected side, and L (left side) is the sham side. α-tubulin was used as a loading control. (**C**) Immunofluorescence staining against actin (phalloidin) and eGFP in the TA muscle; scale bar: 50 μm. (**D**) Hematoxylin and eosin (HE) stain of the TA muscle; scale bar: 50 μm. (E) The twitch force of the isolated EDL muscle of the sham and the AAV sides. The data shown in the same color were from the same mouse. (**F**) The normalized twitch force calculated from E. No significant difference was observed between the sham (36.0 ± 3.01 mN/mm^2^) and AAV sides (35.8 ± 3.25 mN/mm^2^). (**G**) The tetanic force of the isolated EDL muscle of the sham and the AAV sides. The data shown in the same color were from the same mouse. (**H**) The normalized tetanic force calculated from G. No significant difference was observed between the sham (67.8 ± 1.59 mN/mm^2^) and the AAV sides (77.3 ± 11.3 mN/mm^2^).

Next, to examine whether the AAV-control vector could work in the SOD1^G93A^ mice as well as the WT mice, we performed intramuscular injection of the control AAV vector into the right lower limb (AAV side) and of saline into the left lower limb (sham side) of the SOD1^G93A^ mice (Figure 4A). Six weeks after the injection, we examined the expression of eGFP protein in the lower limbs. Immunoblotting of EDL and TA muscle tissues also showed the expression of eGFP in the limb of the AAV6 side but not in the limb of the sham side (Figure 4B). Immunofluorescence staining of the TA muscle tissues showed the expression of eGFP in the limb of the AAV6 side but not in the limb of the sham side (Figure 4C). HE stains of the TA muscle tissues did not show the obvious differences in shapes of cells among the AAV and sham sides (Figure 4D). Thus, we successfully expressed the eGFP proteins in the muscles of lower limbs by intramuscularly injecting the control AAV6 vector in the SOD1^G93A^mice. Next, we investigated the effects of the control AAV6 vector injection on the muscle force production in the EDL muscle of the SOD1^G93A^ mice. The cross-sectional area of isolated EDL muscle showed no significant difference in each side (Supplementary Material). No significant differences in the normalized twitch force were observed between the sham and the AAV sides (36.0 ± 3.01 mN/mm^2^ vs. 35.8 ± 3.25 mN/mm^2^, respectively, *p* = 0.94, Figure 4E,F). Furthermore, there were no significant differences in the normalized tetanic force between the sham and the AAV sides (67.8 ± 1.59 mN/mm^2^ vs. 77.3 ± 11.3 mN/mm^2^, respectively, *p* = 0.14, Figure 4G,H). In addition, the twitch and tetanic force of the SOD1 mice were much lower than those of the WT mice. These results indicated that the control AAV6 vector injection did not affect the muscle force generation in the SOD1^G93A^ mice.

### 2.5. The Intramuscular Injection of the skMLCK Vector Increases the Muscle Force Produced by Twitch and Tetanic Stimulations in SOD1^G93A^ Mice

**Figure 5:**
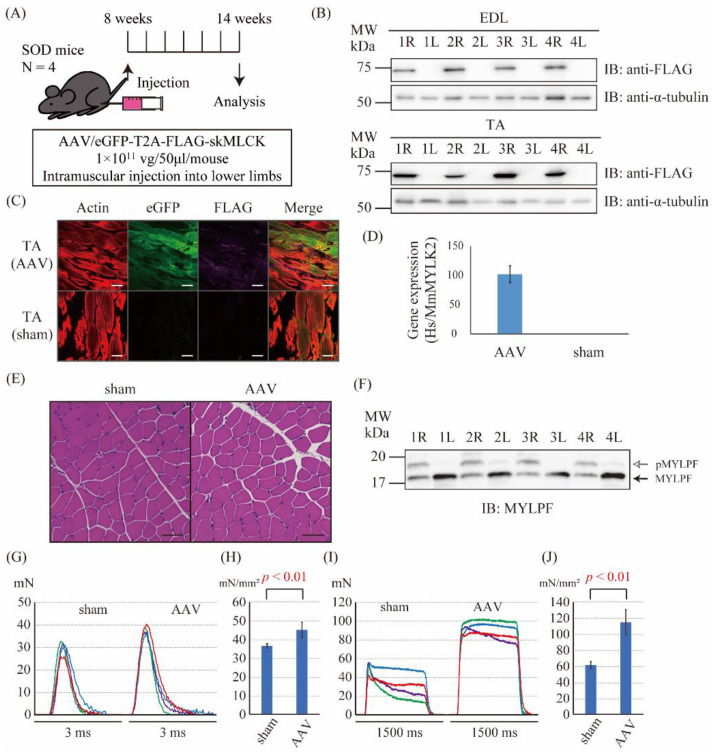
The gene transfer to SOD1 mice using the skMLCK vector. (**A**) The scheme of the injection of the skMLCK vector. The control vector (1 × 10^11^ vg/mouse) was injected into the right lower limb, and saline was injected into the left lower limb at the age of 8 weeks. A series of analysis was performed after 6 weeks. (**B**) Immunoblot analysis of the FLAG peptide in the extensor digitorum longus (EDL) (upper panel) and tibialis anterior (TA) muscles (lower panel). R (right side) is the AAV-injected side, and L (left side) is the sham side. α-tubulin was used as a loading control. (**C**) Immunofluorescence staining against actin (phalloidin), eGFP, and FLAG in the TA muscle; scale bar: 50 μm. (**D**) Gene expression of the human (Hs) *MYLK2* gene in the TA muscle. Gene expression was demonstrated as the ratio of *HsMYLK2* to mouse (Mm) *MYLK2*. (**E**) Hematoxylin and eosin (HE) stain of the TA muscle; scale bar: 50 μm. (**F**) The Phos-tag PAGE of EDL muscle. The upper band showed the phosphorylated MYLPF protein (pMYLPF, white arrow) and the lower band showed the non-phosphorylated MYLPF protein (black arrow). R (right side) is the AAV-injected side, and L (left side) is the sham side. (**G**) The twitch force of isolated EDL muscle of sham and AAV sides. The data shown in the same color were from the same mouse. (**H**) The normalized twitch force calculated from G. The normalized twitch force of the AAV sides (45.1 ± 4.02 mN/mm^2^) was significantly greater than that of the sham sides (36.4 ± 1.22 mN/mm^2^, *p* < 0.01). (**I**) The tetanic force of the isolated EDL muscle of the sham and the AAV sides. The data shown in the same color were from the same mouse. (**J**) The normalized tetanic force calculated from I. The normalized tetanic force of the AAV sides (114.6 ± 16.0 mN/mm^2^) was significantly greater than that of the sham sides (61.2 ± 4.44 mN/mm^2^, *p* < 0.01).

Finally, to examine whether the AAV6-skMLCK vector can improve the force generation in SOD1^G93A^ mice, the AAV6-skMLCK vector was injected intramuscularly into the right lower limb (AAV side) and saline was injected into the left lower limb (sham side) of the SOD1^G93A^ mice (Figure 5A). Six weeks after the injection, we examined the expression levels of mRNA and proteins of eGFP and FLAG-skMLCK in the lower limbs. Immunoblotting of EDL and TA muscle tissues also showed the expression of FLAG proteins in the limb of the AAV6 side but not in the limb of the sham side (Figure 5B). Immunofluorescence staining of the TA muscle tissues showed the expression of eGFP and FLAG-skMLCK proteins in the limb of the AAV6 side but not in the limb of the sham side (Figure 5C). ddPCR showed that the *HsMYLK2* gene expressed only in the AAV-injected sides. The ratio of *HsMYLK2* gene expression to *MmMYLK2* in the AAV side was 102.2 ± 14.6 (Figure 5D, N = 4). HE stains of the TA muscle tissues did not show the obvious differences in shapes of cells among the AAV and sham sides (Figure 5E). Thus, we successfully overexpressed the skMLCK protein in the muscles of the lower limbs intramuscularly injecting the AAV6-skMLCK vector in the SOD1^G93A^ mice. Moreover, the level of the phosphorylated MYLPF was significantly upregulated in the AAV sides by Phos-tag PAGE (Figure 5F). Next, the effect of the AAV6-skMLCK vector injection on the muscle force production in the EDL muscle of SOD1^G93A^ mice was investigated. The cross-sectional area of isolated EDL muscle showed no significant difference in each side (Supplementary Material). The normalized twitch force of the AAV-injected sides was significantly larger than that of the sham sides (45.1 ± 4.02 mN/mm^2^ vs. 36.4 ± 1.22 mN/mm^2^, respectively, N = 4, *p* < 0.01, Figure 5G,H). Furthermore, the normalized tetanic force of the AAV-injected sides was also significantly bigger than that of the sham sides (114.6 ± 16.0 mN/mm^2^ vs. 61.2 ± 4.44 mN/mm^2^, respectively, N = 4, *p* < 0.01, Figure 5I,J). These results indicated that the overexpression of the skMLCK protein by the AAV6-skMLCK vector followed by phosphorylation of MYLPF increased both the twitch and tetanic forces of muscles in SOD1^G93A^ mice.

## 3. Discussion

In this study, the effects of the AAV6 vector carrying the *MYLK2* gene on muscle force development were investigated. Our major findings were as follows: (1) skMLCK was efficiently expressed in EDL and TA muscles by the intramuscular injection of the AAV6-skMLCK vector; (2) the injection of the AAV6-skMLCK vector enhanced the twitch force in SOD1^G93A^ mice but not in WT mice; (3) the injection of the AAV6-skMLCK vector enhanced the tetanic force in both WT and SOD1^G93A^ mice; however, its effects on the muscles of SOD1^G93A^ mice were profound.

Although neuromuscular disease, including ALS, is currently lacking fundamental therapy, several novel potential therapies for neuromuscular diseases are in development. In the case of Duchenne muscular dystrophy, which is caused by mutations in the dystrophin-coded gene, the gene therapies to replace, restore, or exon skipping of the affected gene have been developed and appear to be effective [23,24]. Onasemnogene abeparvovec (Zolgensma, formerly AVXS-101), a gene replacement therapy comprising an AAV vector containing the survival motor neuron 1 (*SMN1*) gene, has also been developed for spinal muscular atrophy type I [25]. Additionally, the intramuscular injection of the AAV9 vector containing laminin subunit alpha 1 (*LAMA1*) gene showed the recovery of muscle function in a mouse model of congenital muscular dystrophy type 1A (MDCA1A), which is caused by the *LAMA2* gene mutation [26], probably due to the compensatory effect of *LAMA1* for *LAMA2* defect. With respect to gene therapy for ALS, the spinal injection of the AAV9 vector encoding shRNA to reduce synthesis of ALS-causing mutant SOD1 was reported in SOD1^G37R^ mice, which demonstrated the improved survival by delaying the onset and slowing the progression of ALS [27]. However, these gene therapies targeting the mutated genes are only used for patients with the mutation but cannot be applied for other gene mutations.

Recently, reldesemtiv, which is a fast skeletal muscle troponin activator and potentially enhances muscle force, was developed as a novel promising drug for the treatment of ALS and is now undergoing clinical trials [28]. This kind of drug has the potential to be broadly used for ALS patients with various mutations because it may be able to increase the force development of the affected muscles by amplifying the Ca^2+^ sensitivity of the thin filaments of the sarcomere in skeletal muscles. Similarly, the other sarcomeric proteins that regulate muscle function can also become the therapeutic targets for ALS. In this study, we focused on RLC phosphorylation by skMLCK. Muscle force is produced by the attachment–detachment cycles between the actin filaments and myosin heads extending from the myosin filament backbone. RLC phosphorylation on the myosin filament induces the myosin head to swing out from a position close to the thick filament’s backbone toward the actin filament, which increases the number of the crossbridges between the myosin and actin filaments, and results in the enhancement of muscle force [17,29]. skMLCK is well-known to modulate skeletal muscle force development by phosphorylating RLC [18]. A series of previous studies have demonstrated that PTP and staircase potentiation were mediated by RLC phosphorylation in skeletal muscles [21,22]. In this study, the strategy of the AAV6 vector encoding skMLCK into skeletal muscles of an ALS mouse model was carried out. Based on the principles of muscle weakness in ALS, in which the motor neurons are primarily affected and followed by muscular dysfunction, skMLCK is not considered to fundamentally amend the muscular dysfunction in ALS. However, we believe that overexpression of skMLCK could improve muscle force generation by amplifying the response of the sarcomeres, similar to reldesemtiv, even with limited neuronal input in ALS patients. Indeed, tetanic forces in WT mice as well as twitch and tetanic forces in SOD1^G93A^ mice were demonstrated to be enhanced by the gene transfer of skMLCK. These results suggested that the increased RLC phosphorylation may improve the tetanic force as well as the twitch force in the affected muscles due to ALS, which was consistent with the previous studies [18,21,22], and gene therapy with the AAV6-skMLCK vector could improve the affected muscle function of patients with various muscular diseases by enhancing the force development. In addition, the gene transfer of skMLCK did not affect the twitch force of WT mice. This result indicated that the overexpression of skMLCK does not affect the twitch force but does affect the tetanic force in healthy conditions. The phosphorylation of RLC is supposed to be related to tetanic force more deeply than twitch force [21]; therefore, the tetanic force of WT mice might be affected more by the overexpressed skMLCK than twitch force even if it is in a healthy condition. Furthermore, the AAV6-skMLCK vector did not fully recover the muscle forces to the level of the WT mice. This may be caused by the difference in the target molecules. In ALS, the motor neurons are primarily affected rather than muscle tissues, although ALS affects muscle function with age [8,30]. The AAV6-skMLCK vector can enhance the developing forces of the muscles but cannot rescue neural degeneration. Therefore, the degree of improvement in muscle forces by skMLCK overexpression may vary depending on the stage of the diseases. In the present study, we did not measure the PTP and staircase potentiation. Because the previous studies reported that the PTP and staircase potentiation are enhanced by the phosphorylation of RLC [21,22], it should be investigated in the future for further confirmation of the effect of the skMLCK overexpression.

In this study, the intramuscular injection of the AAV6 vector was employed, and the significant expression of the target proteins was obtained, as a previous study using intramuscular injection showed no side effects due to local injection, which included inflammation and cellular damage [26]. Furthermore, our data also demonstrated that the control vectors did not show any outstanding diverse effect on the muscles and did not affect the muscle force. Thus, the intramuscular injection procedure itself did not affect the muscle function and was not harmful to the muscles. Generally, the procedure of local injection is much safer and easier than that of systemic injection, such as intravenous and intraspinal. Furthermore, local injection requires a lower concentration of viral vectors than systemic injection, is low cost, and is more accessible by many patients. ALS patients have several difficulties in the movement of their bodies, communication, eating, and breathing at the end stage of ALS, which makes them feel more disabled by the day because their consciousness is usually not affected. Local injection around the limbs or the larynx might improve communication or rescue patients from dysphagia, respectively. However, further examinations are necessary to identify ways of delivery and which serotypes are better for gene therapy of ALS. Finally, the amount of the target gene expression was high in this study, although no harmful effect was found in the muscle tissues examined using HE staining. Therefore, the optimal dose of AAV vector, which can enhance muscle force without harmful effect, should be investigated in the future. Conversely, systemic protein expression is necessary to improve patients’ QOL, because neuromuscular diseases usually show systemic muscular dysfunction. Therefore, a proper injection route should be chosen depending on the situation.

In conclusion, gene transfer therapy of skMLCK using AAV vector could be a new potential therapeutic option for ALS as well as other neuromuscular diseases.

## 4. Materials and Methods

### 4.1. Animals

All animal experiments were performed in accordance with institutional guidelines provided by the Osaka University School of Medicine Animal Care and Use Committee. C57BL/6J mice were purchased from Kiwa Laboratory Animals Co., Ltd. (Wakayama, Japan) and used as wild-type (WT) mice. SOD1^G93A^ mice were purchased from The Jackson Laboratory (B6. Cg-Tg(SOD1-G93A)1Gur/J, 004435, Bar Harbor, ME, USA).

### 4.2. Antibodies

Antibodies used were anti-FLAG antibodies (1:400 dilution for immunostaining, no. 14793; Cell Signaling Technology, Danvers, MA, USA); Alexa Fluor secondary antibodies (1:500 dilution for immunostaining, Thermo Fisher Scientific, Waltham, MA, USA); rhodamine phalloidin (3:500 dilution for immunostaining, PHDR1; Cytoskeleton, Inc., Denver, CO, USA); anti-FLAG^®^ M2-HRP antibody (1:4,000 dilution for immunoblotting, A8592; Sigma–Aldrich, St. Louis, MO, USA); anti-GFP HRP-DirecT antibody (1:2000 for immunoblotting, no. 598-7; MBL, Nagoya, Japan); anti-α-tubulin antibody (1:1000 for immunoblotting, no. B-5-1-2; Abcam, Cambridge, UK); HRP-coupled goat anti-mouse antibody (1:8,000, no. 55550; MP biomedicals, Santa Ana, CA, USA); and anti-skeletal RLC known as MYLPF (1:4000, dilution for immunoblotting, 16052-1-AP; Proteintech, Rosemont, IL, USA).

### 4.3. AAV Vector Construction and Preparation

AAV-6 was selected as a serotype of AAV, which was previously shown to possibly transfer the target gene to skeletal muscles [31]. AAV-6 encoding human skMLCK was prepared using the AAVpro12 Helper Free System (Takara Bio Inc., Shiga, Japan). cDNA fragments encoding human *MYLK2* (gene accession no. NM_033118.4) was PCR amplified using human cDNA template from skeletal muscle. The primers for *MYLK2* were as follows: the forward, 5′-atggcgacagaaaatggagcagttgagctg-3′; and the reverse, 5′-tcagacccccagagccatcagtgcccccga-3′. CMV promotor was employed because of its ability to express a cloned gene in the skeletal muscle via intramuscular injection of AAV [32]. We attached eGFP gene, T2A peptide and FLAG tag in the N terminal of skMLCK, and finally produced the AAV6-eGFP-T2A-FLAG-skMLCK vector (Figure 1A). Furthermore, we also produced the AAV6-eGFP vector as a control vector (Figure 1B). The vector was concentrated using a centrifugal filter (Amicon Ultra-4 100, Millipore, Billerica, MA, USA). The vector titers were determined by real-time PCR, and the final concentration is expressed as the viral genome per milliliter (2 × 10^12^ vg/mL). The viral particles were divided it into small portions and stored at −80 °C until further use.

### 4.4. In Vitro AAV Infections

HEK293 cells (ATCC, Manassas, VA, USA) were cultured in Dulbecco’s modified Eagle’s medium (Thermo Fisher Scientific, Waltham, MA, USA). Twenty-four hours after the seeding of HEK293T cells at a concentration of 4 × 10^4^ cells per well (3.5 mm glass bottom dish for the immunofluorescence staining and 48 well plate for Western blotting), the cells were treated with 200 μL of the culture medium containing AAV vectors at concentrations of 5 × 10^8^–5 × 10^10^ vg/mL. After 48 h, the protein expression was analyzed by the immunofluorescence staining and the Western blotting. For immunofluorescence staining, cells, which were cultured on the glass bottom dish, were immersed in 4% paraformaldehyde (PFA) in 0.1 M phosphate-buffered saline (PBS) for 30 min at room temperature. For Western blotting, cells were lysed in lysis buffer (10 mM Tris–HCl, pH 7.2, 0.15 M NaCl, 1% NP40, and 1 mM EDTA) and separated by sulfate–polyacrylamide gel electrophoresis (SDS–PAGE).

### 4.5. Immunofluorescence Staining

Samples were fixed with 4% PFA in 0.1 M PBS for 30 min at room temperature. Thereafter, they were permeabilized in 0.2% Triton X-100 in PBS for 30 min at room temperature, and were blocked with Dako Protein Block Serum-Free (Agilent, Santa Clara, CA, USA) for 30 min at room temperature. The samples were incubated with primary antibodies in Dako Antibody Diluent (Agilent) overnight at 4 °C. Moreover, the Alexa Fluor secondary antibodies in PBS were incubated with sections for 45 min at room temperature. To stain the F-actin, rhodamine phalloidin was incubated in PBS for 45 min at room temperature. The confocal images were acquired using FV1000 laser scanning microscope (Olympus, Tokyo, Japan) with WHN10x/22 ocular lens and UCPLFLN 100× objective lens (Olympus). The acquisition software was FV10-ASW (Olympus), and Z-projections were processed with FV31S-SW (Olympus).

### 4.6. Sodium Dodecyl Sulfate–Polyacrylamide Gel Electrophoresis

The method of SDS–PAGE was same as the previous article [33]. The stacking gel comprised of 12% acrylamide, 0.1% sodium dodecyl sulfate (SDS), 125 mM Tris–HCl (pH 6.8), 0.1% ammonium persulfate, and 0.5% *N*,*N*,*N*′,*N*′-tetramethylethylenediamine and the resolving gel comprised of 12% acrylamide, 0.1% SDS, 375 mM Tris–HCl (pH 8.8), 0.05% ammonium persulfate, and 0.25% *N*,*N*,*N*′,*N*′-tetramethylethylenediamine were used for SDS–PAGE. Electrophoresis was performed in 0.1% SDS, 25 mM tris, and 192 mM glycine at 180 V per gel for 50 min.

### 4.7. Western Blotting

Proteins were transferred to poly vinylidene difluoride (PVDF) membranes (0.2 μm; Bio-Rad, Hercules, CA, USA) at 15 V for 30 min in transfer buffer. Membranes were incubated with 3% non-fat dry milk in Tris-buffered saline containing Tween [TTBS; 50 mM Tris–HCl (pH 7.8), 150 mM NaCl, and 0.1% Tween 20] for 1 h at room temperature and then in primary antibody in TTBS for 1 h at room temperature followed by secondary antibody in TTBS for 30 min at room temperature. After washing with TTBS (three times for 5 min each), membranes were incubated with the ECL Western Blotting Detection Reagent (GE Healthcare Bioscience, Piscataway, NJ, USA). The emitted light was detected and quantified with a chemiluminescence imaging analyzer, ImageQuant LAS 4000 (GE Healthcare Bioscience, Piscataway, NJ, USA).

### 4.8. In Vivo AAV Injections

At 8 weeks of age, littermate- and copy number-matched mice were randomly divided into AAV6-eGFP-T2A-FLAG-skMLCK and AAV6-eGFP treatment groups. Intramuscular injections with AAV6 vectors (2 × 10^12^ vg/mL, 50 μL, total viral copy was 1 × 10^11^ vg per mice) into the right lower limb and intramuscular injections with 50 μL of saline into the left lower limb (sham) were performed. The dose of viral vector and the method of intramuscular injection were determined based on previous articles [34,35]. After 6 weeks, force measurement of isolated EDL muscle was performed. Harvested tissues of EDL and TA muscle were analyzed by immunofluorescence staining, Western blotting, ddPCR, and HE stain. In previous studies, SOD1^G93A^ mice have shown the phenotype of muscle weakness at around 90 days after birth [8,30]. Therefore, the age of mice for injection was determined in order to show the muscle weakness point at the analysis stage.

### 4.9. Droplet Digital PCR

Total RNA was isolated from homogenized TA muscles using NucleoSpin^®^ (Macherey-Nagel, Düren, Germany). cDNA synthesis was performed using Applied Biosystems ^TM^ High-Capacity cDNA Reverse Transcription Kit (Thermo Fisher Scientific, Waltham, MA, USA). Reverse transcript PCR (RT-PCR) conditions were: 25 °C for 10 min followed by 2 h at 37 °C, and 5 min at 85 °C. Droplet digital PCR (ddPCR ^TM^) was performed on QX200 ^TM^ Droplet Digital PCR system (Bio-Rad, Hercules, CA, USA), using the PrimePCR ^TM^ ddPCR ^TM^ Expression Probe Assay for rat. ddPCR parameters were: 10 min at 95 °C, 40 cycles of 30 s at 94 °C, 1 min at 56 °C, and 10 min at 98 °C. TATA binding protein (Tbp) was used as an endogenous control. Each probe for human *MYLK2* (Unique Assay ID: dHsaCPE5047674, Bio-Rad, Hercules, CA, USA), mouse MYK2 (dMmuCPE5116690, Bio-Rad), and mouse Tbp (dMmuCPE5124759, Bio-Rad) were designed and purchased from Bio-Rad custom order.

### 4.10. Protein Extraction from Muscle Tissues

Muscle samples were homogenized using MULTI BEADS SHOCKER^®^ (Yasui Kikai Corp., Osaka, Japan) in a sample buffer dyed with bromophenol blue (BPB) containing 50 mM Tris-HCl (pH 6.8), 10% grycerol, 1% sodium dodecylsulfate (SDS), and 6% 2-mercaptoethanol (2-ME). After sonication for 3 min and centrifugation at 14,000× *g* for 10 min, supernatants were collected as protein solution of muscle samples.

### 4.11. Cryosections

Muscle tissues for immunofluorescence staining were embedded in an Optimal Cutting Temperature (OCT) matrix compound (Tissue-Tek, Sakura Finetek, Torrance, CA, USA), frozen with dry ice, and mounted in the cryostat. Sections were cut for 20-μm thickness.

### 4.12. Hematoxylin and Eosin Stain

HE stain was performed by Applied Medical Research Laboratory (Osaka, Japan). The slides were observed by optical microscope (BZ-X800; Keyence, Osaka, Japan).

### 4.13. Phos-Tag Sodium Dodecyl Sulfate–Polyacrylamide Gel Electrophoresis

For analysis of RLC phosphorylation by skMLCK, samples were subjected to phosphate affinity SDS–PAGE using an acrylamide-pendant phosphate-binding tag (Phos-tag SDS–PAGE [33]). Phos-tag can capture phosphate monoester dianion and detect the phosphorylated protein [36]. The stacking gel was composed of 12% acrylamide, 0.1% SDS, 125 mM Tris–HCl (pH 6.8), 0.1% ammonium persulfate, and 0.5% *N*,*N*,*N*′,*N*′-tetramethylethylenediamine. The resolving gel was composed of 12% acrylamide, 30 μM Phos-tag acrylamide (Wako, Osaka, Japan), 60 μM MnCl2, 0.1% SDS, 375 mM Tris–HCl (pH 8.8), 0.05% ammonium persulfate, and 0.25% *N*,*N*,*N*′,*N*′-tetramethylethylenediamine. Electrophoresis was performed in 0.1% SDS, 25 mM Tris, and 192 mM glycine at 150 V per gel for 80 min. After electrophoresis, the gel was soaked in EDTA (+) transfer buffer containing 10 mM EDTA, 50 mM Tris, 380 mM glycine, 0.000375% SDS, and 20% EtOH for 10 min and then in transfer buffer for 10 min. Proteins were transferred to PVDF membranes (0.2 μm; Bio-Rad, Hercules, CA, USA) at 15 V for 30 min in transfer buffer. Membranes were then blocked with non-fat dry milk for 1 h at room temperature, incubated with primary antibody in non-fat dry milk for 1.5 h at room temperature followed by secondary antibody in non-fat dry milk for 45 min at room temperature, and detected as with SDS–PAGE. The amount of phosphorylation was analyzed by ImageQuant TL (GE Healthcare, Piscataway, NJ, USA).

### 4.14. Force Measurements of Isolated Extensor Digitorum Longus Muscle

The surgically isolated EDL muscles from mice were mounted on the force transducer (ULA-100GR; MinebeaMitsumi Inc., Nagano, Japan). Muscles were stimulated with two silver chloride electrodes in oxygenated Tyrode’s buffer comprised of 134 mM NaCl, 5 mM KCl, 0.34, 1 mM MgCl2, 5 mM HEPES, and glucose 2 g/L, pH 7.4 at 37 °C. First, we investigated the muscle length for maximal isometric twitch tension (Lo). After adjusting muscle length to 0.7 Lo (length equal to 0.7 of the maximal force development) [21], muscles were stimulated at 0.5 Hz for 2 min as isomeric twitch, followed by 150 Hz for 1 s as tetanus. The voltage of stimulation was set twice as much as the value, which can induce the maximum twitch force. We then recorded the produced force (mN) of twitch and tetanus. The cross-sectional area (mm^2^) of isolated EDL muscle was calculated using the following formula: cross-sectional area = muscle mass/(muscle length × density), the density of muscle was 1.0564 g/cm^3^ [37]. The muscle force was divided by cross-sectional area for normalization and called the normalized force (mN/mm^2^) in the present study.

### 4.15. Statistics

Statistical analyses were performed using the JMP version 14 statistical software (IBM Japan, Tokyo, Japan). All data are expressed as means ± standard deviation (SD). A student’s t-test was performed for the comparison between the sham and AAV injected sides. Values of *p* < 0.05 were considered to represent a significant difference.

## 5. Conclusions

The AAV6/skMLCK vector successfully showed the overexpression of skMLCK in TA and EDL muscles. The control vector did not affect the muscle force in either of the WT or SOD1^G37R^ mice, and the skMLCK vector enhanced the twitch force of the SOD1^G37R^ mice and the tetanic force of the WT and SOD1^G37R^ mice. We conclude that the gene transfer of skMLCK has the potential to rescue weakened muscle function due to various diseases.

## Data Availability

The datasets generated during and/or analyzed during the current study are available from the corresponding authors on reasonable request.

## Supplementary Materials

The following supporting information can be downloaded at: https://www.mdpi.com/article/10.3390/ijms23031747/s1.
