# Supplementary material for: Gene Transfer of Skeletal Muscle-Type Myosin Light Chain Kinase via Adeno-Associated Virus 6 Improves Muscle Functions in an Amyotrophic Lateral Sclerosis Mouse Model
Source: Int J Mol Sci. 2022 Feb 3;23(3):1747. doi: 10.3390/ijms23031747 (PMC8836241; doi:10.3390/ijms23031747)
Supplement: Supplementary file 1 [file ijms-23-01747-s001.zip › ijms-1553423-supplementary.pdf]

International Journal of *Molecular Sciences*

MDPI

Article

Gene Transfer of Skeletal Muscle-Type Myosin Light Chain Kinase via Adeno-Associated Virus 6 Improves Muscle Functions in an Amyotrophic Lateral Sclerosis Mouse Model

Ryohei Oya [1,2], Osamu Tsukamoto [1,*], Tatsuro Hitsumoto [1], Naoya Nakahara [3], Chisato Okamoto [1], Ken Matsuoka [1], Hisakazu Kato [1], Hidenori Inohara [2] and Seiji Takashima [1]

[1] Department of Medical Biochemistry, Osaka University Graduate School of Medicine/Frontier Biosciences, Osaka,565-0871 Japan; roya@ent.med.osaka-u.ac.jp (R.O.); hituhituhitu@gmail.com (T.H.); saiseikaichisato@gmail.com (C.O.); kmatsuoka@medbio.med.osaka-u.ac.jp (K.M.); katohisa@medbio.med.osaka-u.ac.jp (H.K.); takasima@cardiology.med.osaka-u.ac.jp (S.T.)

[2] Department of Otorhinolaryngology Head and Neck Surgery, Osaka University Graduate School of Medicine, Osaka, 565-0871 Japan; hinohara@ent.med.osaka-u.ac.jp

[3] Department of Molecular Physiology, The Jikei University School of Medicine, Tokyo, 105-8461 Japan; nkhr@jikei.ac.jp

* Correspondence: tsuka@madbio.med.osaka-u.ac.jp; Tel.: +81-6-6879-3492; Fax: +81-6-6879-3493

Citation: Oya, R.; Tsukamoto, O.; Hitsumoto, T.; Nakahara, N.; Okamoto, C.; Matsuoka, K.; Kato, H.; Inohara, H.; Takashima, S. Gene Transfer of Skeletal Muscle-Type Myosin Light Chain Kinase via Adeno-Associated Virus 6 Improves Muscle Functions in an Amyotrophic Lateral Sclerosis Mouse Model. *Int. J. Mol. Sci.* **2022**, *23*, 1747. https://doi.org/10.3390/ijms23031747

Academic Editor: Thomas C. Irving

Received: 28 December 2021
Accepted: 1 February 2022
Published: 3 February 2022

Publisher's Note: MDPI stays neutral with regard to jurisdictional claims in published maps and institutional affiliations.

Copyright: © 2022 by the authors. Licensee MDPI, Basel, Switzerland. This article is an open access article distributed under the terms and conditions of the Creative Commons Attribution (CC BY) license (http://creativecommons.org/licenses/by/4.0/).

Table S1. The cross-sectional area of isolated EDL muscle among each experiment.

	**Sham Side**	**AAV Side**	***p* Value**
WT mice for control vector	0.82 ± 0.083 mm^2	0.85 ± 0.033 mm^2	0.32
WT mice for skMLCK vetor	0.84 ± 0.030 mm^2	0.86 ± 0.040 mm^2	0.20
SOD mice for control vector	0.83 ± 0.052 mm^2	0.82 ± 0.039 mm^2	0.20
SOD mice for skMLCK vector	0.83 ± 0.069 mm^2	0.84 ± 0.088 mm^2	0.42

Data was expressed as means ± standard deviation, and analyzed by student’s T test.
